# Abnormal cannabidiol attenuates experimental colitis in mice, promotes wound healing and inhibits neutrophil recruitment

**DOI:** 10.1186/s12950-016-0129-0

**Published:** 2016-07-14

**Authors:** Regina M. Krohn, Sean A. Parsons, Jakub Fichna, Kamala D. Patel, Robin M. Yates, Keith A. Sharkey, Martin A. Storr

**Affiliations:** Division of Gastroenterology, Department of Medicine, University of Calgary, Calgary, AB Canada; Snyder Institute for Chronic Diseases, University of Calgary, Calgary, AB Canada; Department of Physiology and Pharmacology, University of Calgary, Calgary, AB Canada; Department of Biochemistry and Molecular Biology, University of Calgary, Calgary, AB Canada; Hotchkiss Brain Institute, Cumming School of Medicine, University of Calgary, Calgary, AB Canada; Department of Comparative Biology and Experimental Medicine, Faculty of Veterinary Medicine, University of Calgary, Calgary, AB Canada; Department of Biochemistry, Medial University of Lodz, Lodz, Poland; Division of Gastroenterology, Department of Medicine, Ludwig Maximilians University of Munich, Marchioninistrasse 15, 81377 Munich, Germany

**Keywords:** O-1918, TNBS-colitis, Wound healing, Neutrophil recruitment, Cannabinoids, Abnormal cannabidiol

## Abstract

**Background:**

Non-psychotropic atypical cannabinoids have therapeutic potential in a variety of inflammatory conditions including those of the gastrointestinal tract. Here we examined the effects of the atypical cannabinoid abnormal cannabidiol (Abn-CBD) on wound healing, inflammatory cell recruitment and colitis in mice.

**Methods:**

Colitis was induced in CD1 mice by a single intrarectal administration of trinitrobenzene sulfonic acid (TNBS, 4 mg/100 μl in 30 % ethanol) and Abn-CBD and/or the antagonists O-1918 (Abd-CBD), AM251 (CB_1_ receptor) and AM630 (CB_2_ receptor), were administered intraperitoneally (all 5 mg/kg, twice daily for 3 days). The degree of colitis was assessed macro- and microscopically and tissue myeloperoxidase activity was determined. The effects of Abn-CBD on wound healing of endothelial and epithelial cells (LoVo) were assessed in a scratch injury assay. Human neutrophils were employed in Transwell assays or perfused over human umbilical vein endothelial cells (HUVEC) to study the effect of Abn-CBD on neutrophil accumulation and transmigration.

**Results:**

TNBS-induced colitis was attenuated by treatment with Abn-CBD. Histological, macroscopic colitis scores and tissue myeloperoxidase activity were significantly reduced. These effects were inhibited by O-1918, but not by AM630, and only in part by AM251. Wound healing of both HUVEC and LoVo cells was enhanced by Abn-CBD. Abn-CBD inhibited neutrophil migration towards IL-8, and dose-dependently inhibited accumulation of neutrophils on HUVEC.

**Conclusions:**

Abn-CBD is protective against TNBS-induced colitis, promotes wound healing of endothelial and epithelial cells and inhibits neutrophil accumulation on HUVEC monolayers. Thus, the atypical cannabinoid Abn-CBD represents a novel potential therapeutic in the treatment of intestinal inflammatory diseases.

**Electronic supplementary material:**

The online version of this article (doi:10.1186/s12950-016-0129-0) contains supplementary material, which is available to authorized users.

## Background

Cannabis has long been recognized in the Western world for its analgesic, appetite stimulant, antiemetic, muscle relaxant and anticonvulsant properties [1]. Today it is known that plant-derived cannabinoids, or phytocannabinoids, such as Δ^9^-tetrahydrocannabinol (Δ^9^-THC) and cannabinol exert their therapeutic effects through the cannabinoid 1 (CB_1_) and CB_2_ receptors of the endocannabinoid system [2]. Extracts of *Cannabis* have been used as therapeutics in gastrointestinal disorders, but the major pharmacologically active component, Δ^9^-THC has psychotropic properties which limit its suitability as a drug [3].

Aiming to circumvent the psychotropic side effects of cannabis, researchers have focused on the therapeutic potential of non-psychotropic cannabinoids, such as the phytocannabinoid cannabidiol (CBD). CBD has been shown to attenuate experimental colitis in mice, when administered topically or systemically [4]. In addition, the anti-inflammatory and modest antioxidant properties of CBD make it a promising candidate for drug development to target a number of systemic diseases, including rheumatoid arthritis and atherosclerosis [5]. Using CBD as the prototype, synthetic analogs have been developed such as the regio-isomer abnormal cannabidiol (Abn-CBD) and it’s close relative O-1602. These ‘atypical’ cannabinoids lack significant binding affinity to cannabinoid receptors, but act on novel targets such as the orphan receptor GPR55 [6, 7]. It has been demonstrated that CBD and O-1602 protect against experimentally induced colitis in mice, but their mechanisms of action requires further investigation, notably, as the protective properties of O-1602 are also observed in mice lacking the GPR55 gene [8, 9]. They might be conferred by GPR18, another target of O-1602 and the putative receptor of Abn-CBD [10]. While studies have examined the vasodilatory and neuroprotective effects of Abn-CBD [7, 11–13], its potential role in the modulation of gastrointestinal inflammation has not been examined.

The aim of this study was to examine if the CBD analogue Abn-CBD has therapeutic potential in the treatment of gastrointestinal inflammation. We tested the hypothesis that Abn-CBD would reduce intestinal inflammation and accelerate epithelial would healing. We first examined the therapeutic effect of Abn-CBD in a murine model of experimentally induced colitis. The Abn-CBD receptor antagonist O-1918, and the CB_1_ and CB_2_ receptor antagonists, AM251 and AM630, respectively, were employed in order to elucidate, if Abn-CBD effects were CB_1_/CB_2_ dependent or conferred by other receptors. Next we examined the effects of the Abn-CBD on neutrophil recruitment, an important cellular mechanism of intestinal inflammation. Lastly, we studied the impact of Abn-CBD on endothelial and epithelial wound healing in vitro*,* to address a further potential therapeutic mechanism of action [14, 15].

## Methods

### Mice

Male CD1 mice (3 weeks old, weighing ~16 g) were purchased from Charles River (Saint-Constant, Quebec, Canada) and kept in-house for 2 weeks prior to experiments. Mice were housed in plastic sawdust floor cages at constant temperature (22 °C) and a 12:12-h light–dark cycle with access to standard laboratory chow and tap water *ad libitum*. Experimental procedures were approved by the University of Calgary Animal Care Committee and conducted according to guidelines of the Canadian Council on Animal Care.

### Drugs and Pharmacological Treatments

Trinitrobenzene sulfonic acid (TNBS) was purchased from Sigma-Aldrich (Oakville, Ontario, Canada). Abn-CBD, dissolved in methyl acetate, O-1918 (1,3-Dimethoxy-5-methyl-2-[(1R,6R)-3­-methyl-6-(1-methylethenyl)-2-cyclohexen-1-yl]benz-ene), AM630 (6-iodo-2-methyl-1-[2-(4-morpholinyl)ethyl-1H-indol-3-yl](4-methoxyphenyl)methanone) and AM251 (*N*-(Piperidin-1-yl)-5-(4-iodophenyl)­-1-(2,4-dichlorophenyl)-4-methyl-1H-pyrazole-3-carboxamide) were obtained from Tocris Bioscience (Bristol, UK). Because of its toxicity, methyl acetate was evaporated prior to the in vivo experiments and ethanol was used instead as a solvent. Abn-CBD was then further diluted in Tween 80 (10 %) and sterile saline. Vehicle consisted of ethanol, Tween 80 and sterile saline (1:1:8). AM630 and AM251 were dissolved in dimethyl sulfoxide (DMSO, 99.7 %) and further diluted with vehicle. 45 min prior to the induction of TNBS colitis, mice were injected intraperitoneally (i.p.) with 5 mg/kg AM630, AM251, O-1918 or vehicle, followed by 5 mg/kg Abn-CBD, 15 min later. As a single dose of Abn-CBD was ineffective (preliminary data not shown), mice were injected twice daily for 3 days. For in vitro assays, 10 mM stock solutions (in DMSO) of Abn-CBD and the CB receptor antagonists were prepared.

### Induction of TNBS colitis

The TNBS colitis experiment was used as an established model for testing cannabinoid effects on experimental colitis [4, 9, 16]. TNBS colitis was induced in male CD1 mice as described previously [17]. Briefly, animals were lightly anaesthetized with isoflurane and TNBS (4 mg in 100 μL of 30 % ethanol) was infused into the colon through a catheter, 1 mm in diameter, inserted 3 cm proximally to the anus in mice. Vehicle alone (100 μL of 30 % ethanol) was administered in control experiments. The mice were weighed daily.

### Macroscopic scoring and damage assessment

Four days after the induction of colitis, mice were euthanized by cervical dislocation. The colon was immediately removed, opened longitudinally along the mesenteric border, and examined. Colonic damage was assessed by a semi-quantitative scoring system as previously described [9]. Macroscopic damage was scored according to the following scale, adding individual scores for ulcer, colonic shortening, wall thickness, and presence of hemorrhage, fecal blood, or diarrhea. Ulcer: 1 point for each 0.5 cm; shortening of the colon: 1 point = >15 %, 2 points = >25 % (based on a mean length of the untreated colon of 7.6 ± 0.55 cm; *n* = 5-10); wall thickness measured in mm. The presence of hemorrhage, fecal blood, or diarrhea increased the score by 1 point for each additional feature. Adhesion of the colon to organs was scored as follows: 1 point = 1 adhesion, 2 points = 2 or more adhesions or adhesions to organs. A new batch of TNBS was used for the experiments with the AM630 and AM251 inhibitors, likely being the reason for the higher overall damage score in these experiments.

### Myeloperoxidase activity

Measurement of myeloperoxidase (MPO) activity in tissue samples was used to assess the degree of granulocyte infiltration [18]. Samples of mouse colon were weighed, immediately frozen on dry ice, and stored at - 80 °C. For determination of MPO activity, the frozen tissue was placed in 0.5 % HTAB buffer (50 mg of tissue/mL; pH 6.0) and disrupted with a Polytron homogenizer (Brinkman Instruments, Mississauga, Ontario, Canada). The detergent HTAB (hexadecyl-trimethyl-ammoniumbromide; Sigma-Aldrich) releases MPO from the primary granules of neutrophils and enhances enzyme activity through the presence of bromide. Afterwards, the homogenate was centrifuged for 15 min at maximum speed and 4 °C. Before reading MPO activity, 7 μL of supernatant was added to 200 μL of 50 mM potassium phosphate buffer (pH 6.0) containing 0.167 mg/mL of O-dianisidine hydrochloride and 0.5 μL of 1 % H_2_O_2_/mL. The kinetics of MPO activity was measured at 460 nm (Thermo Fischer Labsystems Multiskan, Thermo Scientific, Ottawa, Ontario, Canada). A mean was calculated for the vehicle-treated group and set at 100 %. Values of all other treatment groups are expressed as percent of the respective vehicle-treated group (TNBS only).

### Histology

For microscopic scoring, segments of the distal colon were stapled flat onto cardboard with the mucosal side up and fixed for 24 h in 10 % neutral-buffered formalin. Tissue was then dehydrated, embedded in paraffin, and standard hematoxylin/eosin staining was performed on 5-μm thick sections. Five sections at least 50 μm apart per colon were scored as described before [19]. Mucosal architecture, muscle thickness, leukocyte infiltration were each scored with 0–3 points where 0 depicts normal colon and 3 the maximally affected colon. The absence of goblet cells scored 1 point. The total score index for one colon was the sum of these subscores and had a maximum of 9.

### Neutrophil isolation and HUVEC culture

Neutrophils were isolated as previously described [20]. Briefly, blood from healthy adult donors was drawn into a heparinized syringe, and allowed to settle on half of its volume of 6 % dextran for 1 h at room temperature. Erythrocytes were removed by hypotonic lysis, and granulocytes were further purified by density centrifugation on lymphoprep 1077. Endothelial cells (HUVEC) were isolated from human umbilical cords (Foothills Hospital, Calgary, Canada) as previously described [21] and maintained in M199 medium (Invitrogen, Carlsbad, CA) containing 20 % human serum. Only the first passage of cells was used for the experiments. All procedures requiring human subjects were approved by The University of Calgary Conjoint Health Research Ethics Board. Study participants provided written consent for donation of their biological samples for research. The colonic epithelial cell line LoVo was purchased from ATCC (# CCL-229) and cultured in F-12 K Medium (ATCC) supplemented with 10 % fetal bovine serum.

### Chemotaxis assay

2.5 × 10^5^ neutrophils were incubated with Abn-CBD and/or O-1918 at the concentrations indicated for 30 min and pipetted into the top chamber of 5 μm pore Transwells® (Corning Life Sciences, Union City, CA). Cells were allowed to migrate through the pores to the lower chamber of the wells containing the chemokine IL-8 at the effective concentration of 10nM. This suboptimal IL-8 concentration was chosen so that that anything that interfered with IL-8’s ability to induce a response could be observed. A random migration control (no stimulus) was run in every experiment. The experiment was stopped after 3 h. Three non-overlapping digital images of the bottom of each chamber were taken using a light microscope, and adherent cells were counted.

### Neutrophil recruitment under flow conditions

Interactions between endothelial cells and freshly isolated human neutrophils were examined under flow conditions. A parallel plate flow chamber from Glycotech (Gaithersburg, MD, USA) was used to mimic physiologic flow conditions.

Neutrophil accumulation and transmigration were determined as previously described for eosinophils [20]. Briefly, endothelial cell monolayers were incubated with 10 ng/ml of TNF-α for 4 h. Either the monolayer was also incubated with Abn-CBD or O-1918 for 4 h, or neutrophils were treated with the drugs 1 h prior to the experiment at indicated concentrations. After TNF-α stimulation, neutrophils (1 × 10^6^/mL) were perfused across the monolayer at 1 dyn/cm^2^ using the parallel plate flow chamber. After 4 min of perfusion, the inlet line was transferred to Hank’s balanced salt solution (HBSS), to prevent the binding of new neutrophils, and buffer was perfused for an additional 4 min. Interactions between neutrophils and endothelial cells were visualized on a Zeiss Axiovert 100 microscope using either a 10X/0.25NA or 40X/0.60NA phase-contrast objective and recorded via a charge-coupled device camera (KP-M1U; Hitachi Denshi, Ltd.). The total number of cells accumulated on the monolayer was determined between 4 and 5 min of perfusion and the number of transmigrated cells were determined between 6 and 7 min. 4–10 fields of view were examined for each condition.

### Immunohistochemistry

Neutrophils on glass coverslips were fixed with 2 % paraformaldehyde in HBSS, then washed with HBSS and blocked for 1 h with 1 % human serum albumin in HBSS. Cells were stained with anti-GPR18 antibody or isotype control for 1 h (1:300 in HBSS), then washed and incubated with the secondary FITC-conjugated detection antibody (1:1000) for 1 h. Cells were mounted with Hoechst 33258 (Sigma-Aldrich, Oakville, Ontario, Canada).

### Scratch wound healing assay

Single-path wound healing experiments were performed as described [22]. Briefly, LoVo cells and HUVEC were grown to confluence in 6-well dishes. A day prior to the experiment cells were starved with serum-deprived medium or medium with low serum content (for HUVEC). Wounds were made by dragging a sterile 10 μl pipette tip across the monolayer to create a cell-free path ~1 mm wide, and cells were incubated with Abn-CBD and/or O-1918 at the concentrations indicated for 20 h, or at 1 μM, where there is no indication. Pictures of the wounds at 0 h and 20 h were taken using an inverted microscope with mounted camera at x100 magnification (Olympus, Center Valley, PA), and quantification of cell migration into the wounded area was done using the MetaMorph® Analysis Software. 3-4 fields of view were analyzed per well.

### Statistical analysis

For statistical analysis a Student’s *t*-test or one-way ANOVA with Tukey’s post-hoc test was performed using the GraphPad Prism software (La Jolla, CA, USA). Significance was set at *p* ≤ 0.05. Data are presented as mean values ± the standard error of the means (SEM).

## Results

### Abn-CBD attenuates the degree of colitis

**Fig. 1:**
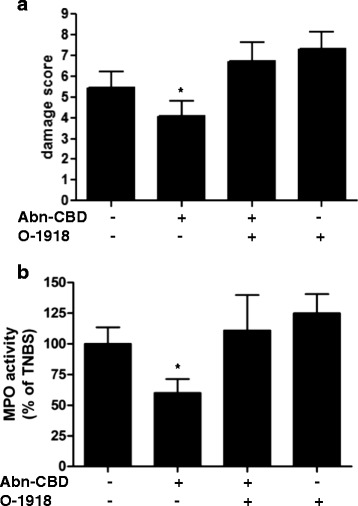
The effects of Abn-CBD treatment on macroscopic damage score and MPO activity. TNBS-treated mice were given O-1918 (5 mg/kg) and/or Abn-CBD (5 mg/kg) twice daily for 3 days. Macroscopic damage score (**a**) and myeloperoxidase activity (**b**) were determined at the end of the experiment, 24 h after the last dose of drug. Abn-CBD significantly reduced macroscopic damage and MPO activity, and effect that was blocked by O-1918. Data were analyzed with one-way ANOVA with Tukey’s post-hoc test. *, *p* < 0.05 when compared to control and Abn-CBD + O-1918 (**a** and **b**) and when compared to O-1918 (**b**); *n* ≥ 10/group

Four days after the induction of colitis, mice showed severe macroscopic colonic damage and inflammation (Fig. 1a). This was reduced by treatment with Abn-CBD (5 mg/kg, i.p., administered twice a day), (Fig. 1a). TNBS-induced weight loss during the experiment was not altered by administration of Abn-CBD (Additional file 1: Figure S1). MPO activity was significantly higher in colitic mice compared to controls and Abn-CBD treated colitic mice (Fig. 1b), indicating that Abn-CBD either inhibits inflammatory neutrophil recruitment or accelerates clearance of neutrophils from the tissue, thus reducing the inflammatory response. The selective Abn-CBD antagonist O-1918 (5 mg/kg) was injected 30 min before treatment with Abn-CBD. O-1918 does not bind to either CB_1_ or CB_2_ receptors, but has been shown to inhibit Abn-CBD induced vasorelaxation through one or more GPCR [23]. O-1918 administration inhibited the beneficial effects of Abn-CBD on macroscopic damage (Fig. 1a). Interestingly, O-1918-treated mice exhibited even greater colon damage than control mice. The same inhibitory effect of O-1918 was observed when examining MPO activity (Fig. 1b). Abn-CBD + O-1918 administration resulted in MPO levels similar to the colons of untreated colitic mice. Again, MPO activity was slightly higher in O-1918 treated groups than in the untreated group.

### Protective effects of Abn-CBD in TNBS-induced colitis are CB_1_/CB_2_ independent

**Fig. 2:**
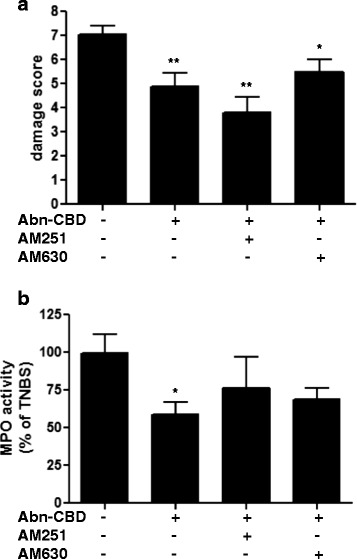
The effects of Abn-CBD treatment in the presence of CB antagonists on macroscopic damage score and MPO activity. TNBS-treated mice were given AM251, AM630 and/or Abn-CBD (all at 5 mg/kg) twice daily for 3 days. Macroscopic damage score (**a**) and myeloperoxidase activity (**b**) were determined at the end of the experiment, 24 h after the last dose of drug. Abn-CBD treatment and Abn-CBD + AM251 or AM630 significantly decreased the damage score. Myeoloperoxidase activity was significantly decreased with Abn-CBD. Data were analyzed with one-way ANOVA with Tukey’s post-hoc test *, *p* < 0.05 and **, p < 0.01 when compared to controls; *n* = 4-5/group

In order to investigate if the effects of abn-CBD were mediated by CB receptors, Abn-CBD was next tested in conjunction with AM251 and AM630, which are selective inverse agonists of CB_1_ and CB_2_, respectively. Treatment of mice with AM251 or AM630 30 min prior to the injection of Abn-CBD did not alter the protective properties of the latter, as assessed macroscopically (Fig. 2a). The reduction of MPO activity caused by Abn-CBD was not affected by the CB_2_ antagonist AM630 (Fig. 2b), but no significant change in MPO activity were seen in colons of mice treated with AM251, when compared to Abn-CBD or control (Fig. 2b).

**Fig. 3:**
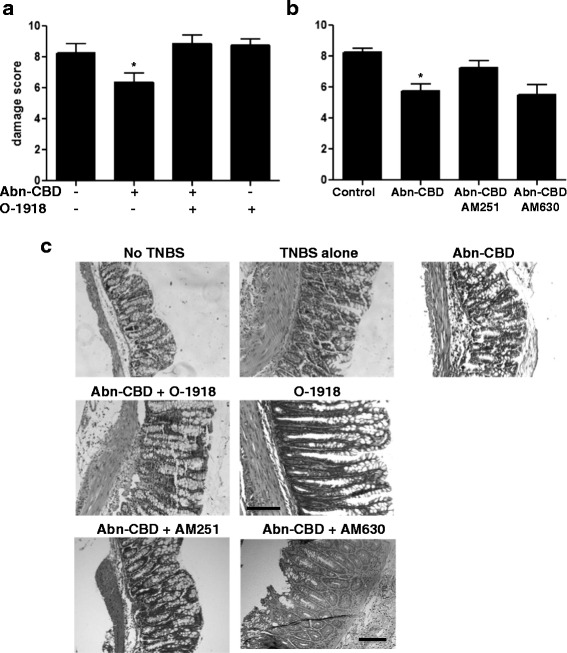
The effects of Abn-CBD treatment on microscopic damage score and histology. TNBS-treated mice were given O-1918, AM251, AM630 (all at 5 mg/kg) and/or Abn-CBD (5 mg/kg) twice daily for 3 days. Microscopic damage score (**a** and **b**) and histological appearance of the hematoxylin/eosin stained tissue (**c**) were determined at the end of the experiment, 24 h after the last dose of drug. TNBS-treated mice displayed severe mucosal damage as well as an increase in lymphocyte and neutrophil infiltration. **a** Mucosal damage was significantly reduced by Abn-CBD treatment and reversed by addition of O-1918; *, *p* < 0.05, when compared to any other treatment group. **b** Mucosal damage was significantly reduced by Abn-CBD treatment and partly reversed by administration of AM251; *, *p* < 0.05, when compared to TNBS alone. Data were analyzed with one-way ANOVA with Tukey’s post-hoc test, *n* = 4-5/group. Scale bar in C. = 100 μm

The microscopic colitis score was significantly lower in mice treated with Abn-CBD, and the protective effect was inhibited by addition of O-1918 (Fig. 3a). Histological sections of the colonic epithelium from Abn-CBD treated mice displayed partially preserved crypt morphology (Fig. 3c), whereas O-1918 with or without Abn-CBD increased abnormal mucosal architecture, lacking most of the goblet cells. Abn-CBD improved the histological appearance (Fig. 3b). This effect was reduced by pretreatment with AM251, while AM630 was unable to reverse the effects of Abn-CBD (Fig. 3b and c).

### Abn-CBD inhibits inflammatory neutrophil recruitment

**Fig. 4:**
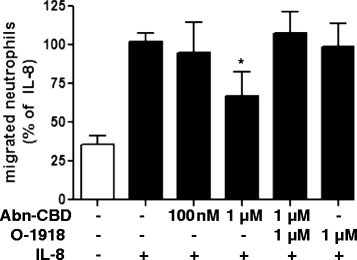
The effects of Abn-CBD on neutrophil chemotaxis. Neutrophils were incubated with Abn-CBD and/or O-1918 and pipetted into the top chamber of 5 μm Transwells (2 × 10^5^/well). Migration towards IL-8 (10 nM) was assessed after 3 h by counting adherent neutrophils at the bottom of the chamber (3 non-overlapping fields/well). The open bar depicts random migration without an IL-8 stimulus. Abn-CBD significantly reduced neutrophil chemotaxis, an effect that was blocked by O-1918. Data were analyzed with one-way ANOVA with Tukey’s post-hoc test. *, *p* < 0.05 when compared to control*; n* = 3/group

Neutrophil infiltration is a prominent feature of inflammatory diseases [24–28], hence experiments were performed to investigate the effect of Abn-CBD on neutrophil recruitment. First, the effect of Abn-CBD on neutrophil chemotaxis towards the chemokine IL-8 was examined. Incubation of freshly isolated human neutrophils with 1 μM Abn-CBD significantly reduced their migration towards 10 nM IL-8, and co-incubation with O-1918 (1 μM) restored the chemotactic activity (Fig. 4). This result demonstrated a direct effect of Abn-CBD on neutrophils.

**Fig. 5:**
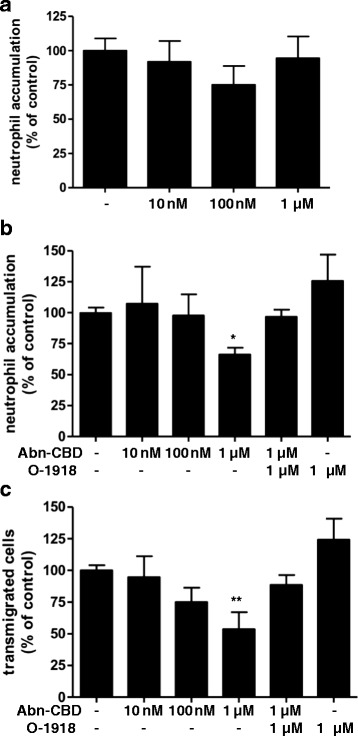
The effects of Abn-CBD on neutrophil recruitment assessed using a parallel plate flow chamber assay. HUVEC monolayers were activated with TNF-α (10 nM) 4 h prior to the experiment. **a** Abn-CBD was added to the HUVEC for 1 h and freshly isolated neutrophils were perfused over the monolayer for 4 min. **b** Using a different approach neutrophils were treated with Abn-CBD and O-1918 for 15 min and then perfused over the HUVEC monolayer, neutrophil accumulation was then assessed after 4 min of perfusion, and **c** transmigration of neutrophils through the monolayer was determined after 6 min. Neutrophil accumulation (after 4 min) and transmigration (after 6 min) was significantly decreased, when neutrophils were incubated with Abn-CBD. *, *p* < 0.05 and **, *p* < 0.01 when compared to control, Abn-CBD + O-1918 and O-1918; *n* = 5/group

**Fig. 6:**
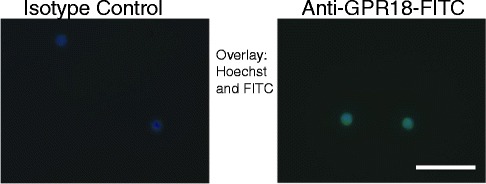
Fluorescence micrographs of GPR18 immunoreactivity. Neutrophils on glass coverslips were fixed with 2 % paraformaldehyde in HBSS and stained with anti-GPR18 antibody (*right panel*) or isotype control (*left panel*), then washed and incubated with the secondary FITC-conjugated detection antibody (*green*). Cells were mounted with Hoechst 33258 (Sigma-Aldrich, Oakville, Ontario, Canada) (*blue*). No immunoreactivity was detected in the control cells, but most neutrophils expressed GPR18. Scale bar = 100 μm

Using flow chambers with confluent HUVEC monolayers, inflammatory neutrophil recruitment was assessed under physiological flow conditions. This experimental set-up can be used to mimic the extravasation of neutrophils from the blood stream into the inflamed tissue. It also allows visualization of the different stages of neutrophil recruitment- rolling, adhesion, and transmigration - which all require distinct and overlapping molecular signalling events [29]. In this experiment, accumulation of neutrophils on inflamed endothelium, i.e. the sum of slow-rolling and firmly adhered neutrophils, was assessed, as well as the number of transmigrated neutrophils. The incubation of the TNF-α stimulated HUVEC monolayer with Abn-CBD (10 nM to 1 μM) did not alter the accumulation of neutrophils (Fig. 5a). However, when neutrophils were incubated with Abn-CBD (1 μM) 15 min prior to perfusion over the monolayer, cell accumulation was significantly decreased (Fig. 5b). Moreover, this inhibitory effect of Abn-CBD was antagonized by O-1918 (1 μM). The same was true for the transmigration rate of neutrophils (Fig. 5c). Fewer transmigrated cells were observed, when neutrophils were pre-treated with Abn-CBD (1 μM), while addition of O-1918 restored the neutrophil transmigration rate to control values. Abn-CBD effects may be conferred by the putative Abn-CBD receptor GPR18, which is expressed in these cells as shown by immunohistochemistry (Fig. 6).

### Abn-CBD promotes wound healing in endothelial and colonic epithelial cells

Endothelial and epithelial integrity is crucial for preserving vascular and intestinal homeostasis [30, 31]. IBD is characterized by ulceration and impairment of the intestinal epithelial barrier [32], and cannabinoids have been shown to promote wound healing of epithelial cells in a CB_1_ dependent manner [33]. Therefore we tested if Abn-CBD could promote epithelial and endothelial wound healing.

**Fig. 7:**
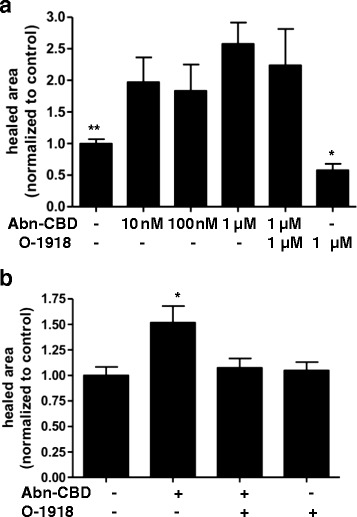
The effects of Abn-CBD treatment on wound healing. HUVEC and LoVo cells were grown to confluence in 6 well plates. Then a scratch wound was made with a pipet tip. **a** HUVEC cells were incubated with the respective concentrations of Abn-CBD and O-1918. Wound area size was measured at 0 and 20 h. Abn-CBD significantly accelerated wound closure in HUVEC. Additional treatment with the O-1918 did not reverse this effect. **b** For incubation of LoVo cells, the effective dose for Abn-CBD (1 μM) was used. Wound area was measured at 0 and 20 h. Abn-CBD significantly accelerated wound closure. The Abn-CBD effect was reversed by addition of the antagonist O-1918. Data were analyzed with one-way ANOVA with Tukey’s post-hoc test. *, *p* < 0.05 when compared to any other treatment; *n* = 5-7/group

**Fig. 8:**
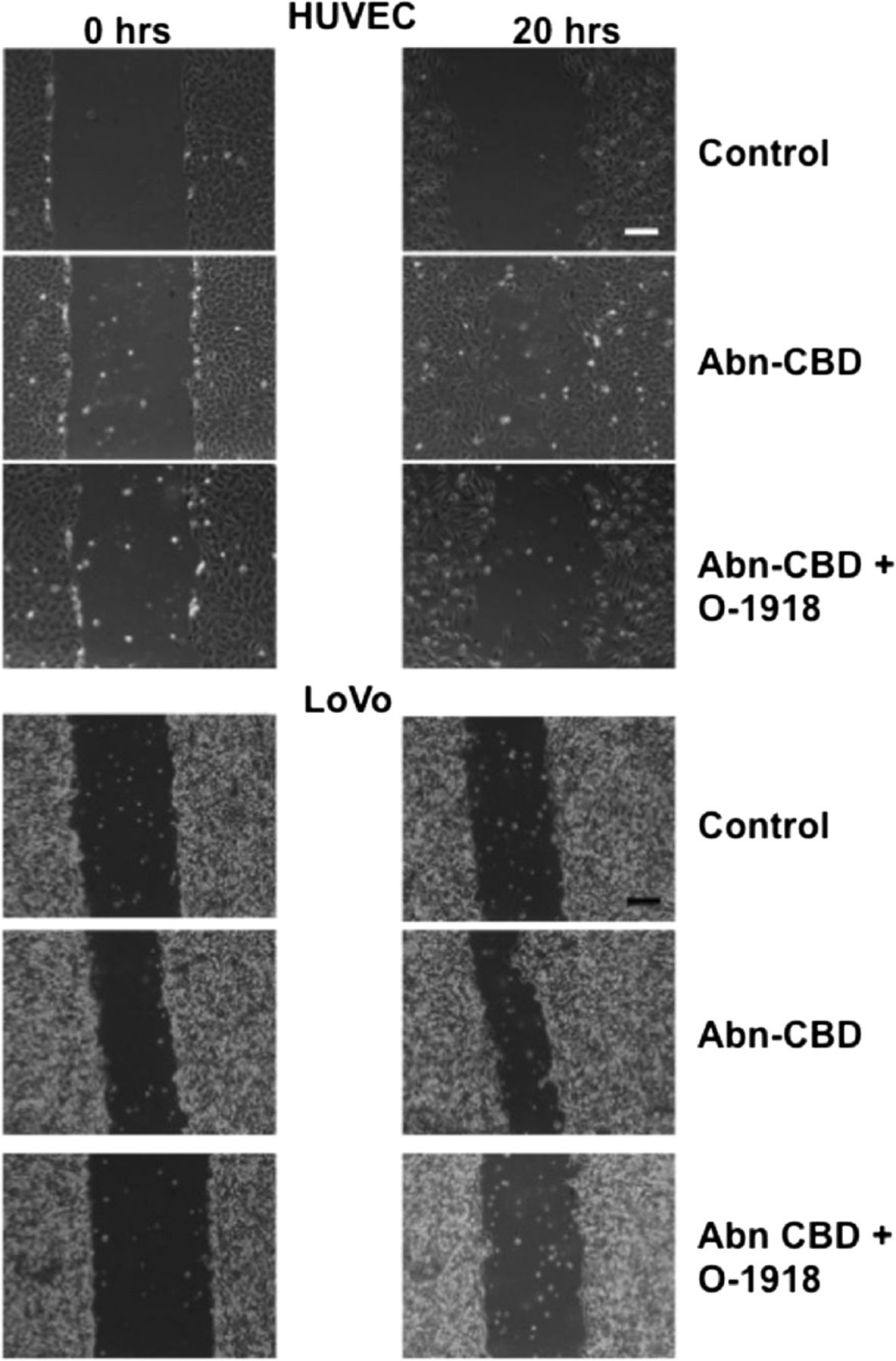
The effects of Abn-CBD treatment on wound healing. HUVEC and LoVo cells were grown to confluence in 6 well plates. Then a scratch wound was made with a pipet tip. Wound area was measured at 0 and 20 h. Scale bar = 100 μm. Abn-CBD treatment accelerated wound healing and O-1918 addition reversed the Abn-CBD effect (see Fig. 7 for detailed results)

Treatment of wounded, serum-deprived HUVEC and LoVo colonic epithelial cells with Abn-CBD caused migration of cells into the wounded area, which was most pronounced at the highest used concentration of 1 μM (Figs. 7a and 8). Abn-CBD wound healing in HUVEC was not significantly inhibited by the addition of O-1918 (Fig. 7a). In LoVo epithelial cells, addition of O-1918 significantly inhibited the Abn-CBD induced cell migration, (Figs. 7b and 8).

## Discussion

The incidence and prevalence of ulcerative colitis and Crohn’s disease is increasing over time and in different regions of the world, suggesting that IBD is an emerging global disease [34]. Numerous studies have revealed that endocannabinoid levels, and the expression of CB_1_ and CB_2_ receptors are increased in IBD patients and that the endocannabinoid system may play a protective role in the development of colitis [35, 36]. Moreover, other studies suggest that cannabinoids may exert anti-inflammatory effects in cardiovascular disease (reviewed in [37]), the number one cause of death globally [38]. Therefore, cannabinoids that exert effects through the endocannabinoid system, but also through novel receptors may have therapeutic potential in inflammatory diseases, especially if the psychotropic properties can be eliminated.

In these studies we examined the therapeutic potential of the non-cannabinoid CBD analogue Abn-CBD for the treatment of inflammatory conditions of the gastrointestinal tract. Here we show that Abn-CBD treatment resulted in a 20–30 % reduction of TNBS-induced colitis in mice, hence Abn-CBD is less effective than previously studied cannabinoid receptor agonists and the atypical cannabinoid O-1602, which all reduced TNBS-induced colitis by 35–55 % [9, 17].

The beneficial effects of Abn-CBD were completely inhibited by O-1918, the antagonist for the putative Abn-CBD receptor, suggesting this was a receptor-mediated effect. Interestingly, O-1918 administration alone increased colitis scores and MPO activity beyond that of controls, which suggests that the actions of an endogenously active anti-inflammatory endocannabinoid were inhibited by O-1918. A previous study showed that O-1918 inhibits the activation of an “endothelial anandamide receptor”, and thus counteracting anandamide-induced vasorelaxation [23]. It has also been demonstrated that anandamide is protective in experimental colitis, and that endogenous levels of anandamide are elevated in IBD patients [39]. Therefore it seems likely that the administration of O-1918 not only blocked the effects of Abn-CBD, but also the effects of anandamide, causing more severe colon damage. We investigated the potential role of CB receptors in the mechanism of action of Abn-CBD. Neither CB_1_ nor CB_2_ receptor antagonists reversed the action of Abn-CBD when assessed at the macroscopic level. Previously it has been shown that CB_2_ receptor inhibition by AM630 exacerbates experimentally induced colitis [17]. It seems that Abn-CBD treatment can compensate for the CB_2_ receptor inhibition by AM630, as the protective effect of Abn-CBD was not reduced by AM630 administration. The microscopic damage assessment revealed a slight inhibitory effect of AM251, which binds to CB_1_ and thereby inhibiting the receptor’s activation [40]. AM251 has also been described to activate GPR55 [41], which is also a ligand for Abn-CBD, though Abn-CBD has a much higher EC_50_ for that receptor [6]. Therefore, a subtle Abn-CBD effect conveyed by GPR55 may be antagonized by AM251.

In order to elucidate the effects of Abn-CBD we assessed wound healing and neutrophil recruitment. These experiments are important as endothelial dysfunction, delayed wound healing and neutrophil infiltration are key features of chronic inflammatory diseases, such as cardiovascular disease and IBD [24, 42–45]. Abn-CBD increased the wound healing capacity of HUVEC. This protective effect was slightly inhibited by the antagonist O-1918. Abn-CBD-induced wound healing of the colonic epithelial cell line LoVo was completely inhibited by O-1918, suggesting that Abn-CBD acts, at least partly through a different, O-1918-independent receptor in the endothelial cells.

In experiments to investigate the effects of Abn-CBD on neutrophil chemotaxis, IL-8 was used as neutrophil attractant, as this chemokine is highly expressed in the inflamed mucosa of IBD patients and also in uninflamed specimens from Crohn’s disease patients [46, 47]. The inhibitory effect of Abn-CBD on neutrophil chemotaxis was completely reversed by the antagonist O-1918 not only in the static chemotaxis assay, but also in a more physiologic neutrophil recruitment assay under flow conditions. Here, neutrophil activation and arrest triggered by inflamed endothelium was inhibited, when neutrophils were pre-treated with Abn-CBD, but not when the HUVEC monolayer was pre-treated with Abn-CBD. Abn-CBD had no significant effect on the pro-inflammatory actions of TNF-α on the endothelium. The process of neutrophil extravasation from the blood stream to sites of inflammation involves the rolling of the cells on the endothelium, firm arrest of the neutrophil, and transmigration through the endothelium. These particular steps require intricate overlapping and distinct molecular events to take place, such as activation of integrins, and increased expression of adhesion molecules and chemokines [29]. In summary, rolling of neutrophils on the endothelium is selectin-mediated and followed by chemokine-triggered activation of leukocytes and their integrin-dependent arrest. Chemokines that lead to activation and adherence of neutrophils are produced by the inflamed endothelium and immobilized on the endothelial cell-surface [48]. The last step of the extravasation process, the transmigration or diapedesis of the neutrophil through the endothelium, also involves redistribution of integrin-adhesion molecule clusters [49]. As incubation of the HUVEC monolayer did not alter neutrophil accumulation, but treatment of neutrophils did, we reason that Abn-CBD interferes with the chemokine-mediated inside-out signaling in neutrophils through GPCRs, which would otherwise lead to an increase of affinity and avidity of integrins. This assumption concurs with the fact that neutrophil rolling (selectin-dependent) was not affected by Abn-CBD (data not shown), but transmigration (integrin-dependent) was. The identification of the inhibitory pathway and identification of the affected integrin-ligand interactions go beyond the scope of this study, but we speculate that Abn-CBD effects may be conferred by GPR18, the putative Abn-CBD receptor [10], as its expression on these cells was confirmed by immunohistochemistry. Furthermore the inhibition of neutrophil chemotaxis by other endocannabinoids were shown to be independent of CB_1_ and CB_2_ receptors [50].

## Conclusion

This study demonstrates that Abn-CBD promotes epithelial, and endothelial wound healing in vitro. Abn-CBD drastically interferes with the inflammatory recruitment of neutrophils in vitro, and in vivo possibly through targeting the receptor. GPR18 is a strong candidate involved in the mechanism by which Abn-CBD improves the outcome of colitis, since attenuation of TNBS-induced colitis by Abn-CBD was not reversed by CB_1_/CB_2_ antagonists on a macroscopic level. These studies point to the potential of non-psychotropic cannabinoids as potential therapeutics for the treatment of IBD, but may also be of importance in other chronic inflammatory diseases like cardiovascular disease, as suggested by Abn-CBD’s strong effect on endothelial cells.

## Abbreviations

Abn-CBD, abnormal cannabidiol; AM251, *N*-(Piperidin-1-yl)-5-(4-iodophenyl)­-1-(2,4-dichlorophenyl)-4-methyl-1H-pyrazole-3-carboxamide; AM630, 6-iodo-2-methyl-1-[2-(4-morpholinyl)ethyl-1H-indol-3-yl](4-methoxyphenyl)methanone; CB_1_, cannabinoid receptor 1; CB_2_, cannabinoid receptor 2; CBD, cannabidiol; DMSO, dimethyl sulfoxide; GPCR, G-protein-coupled receptor; IBD, inflammatory bowel disease; MPO, myeloperoxidase; O1918, 1,3-Dimethoxy-5-methyl-2-[(1R,6R)-3­-methyl-6-(1-methylethenyl)-2-cyclohexen-1-yl]benz-ene; TNBS, trinitrobenzene sulfonic acid

## Additional file

Additional file 1: Figure S1 Weight loss in animals with colitis. O-1918 (5 mg/kg) and/or Abn-CBD (5 mg/kg) were given twice daily for 3 days to TNBS-treated mice. Weight of the animals was recorded daily. All animals lost weight throughout the duration of the experiment. Abn-CBD treatment did not decrease weight loss. No significant differences in weight loss were observed. (PPTX 3237 kb)
