# Supplementary figures and images for: Abnormal cannabidiol attenuates experimental colitis in mice, promotes wound healing and inhibits neutrophil recruitment
Source: J Inflamm (Lond). 2016 Jul 14;13:21. doi: 10.1186/s12950-016-0129-0 (PMC4944257; doi:10.1186/s12950-016-0129-0)

## Slide 1
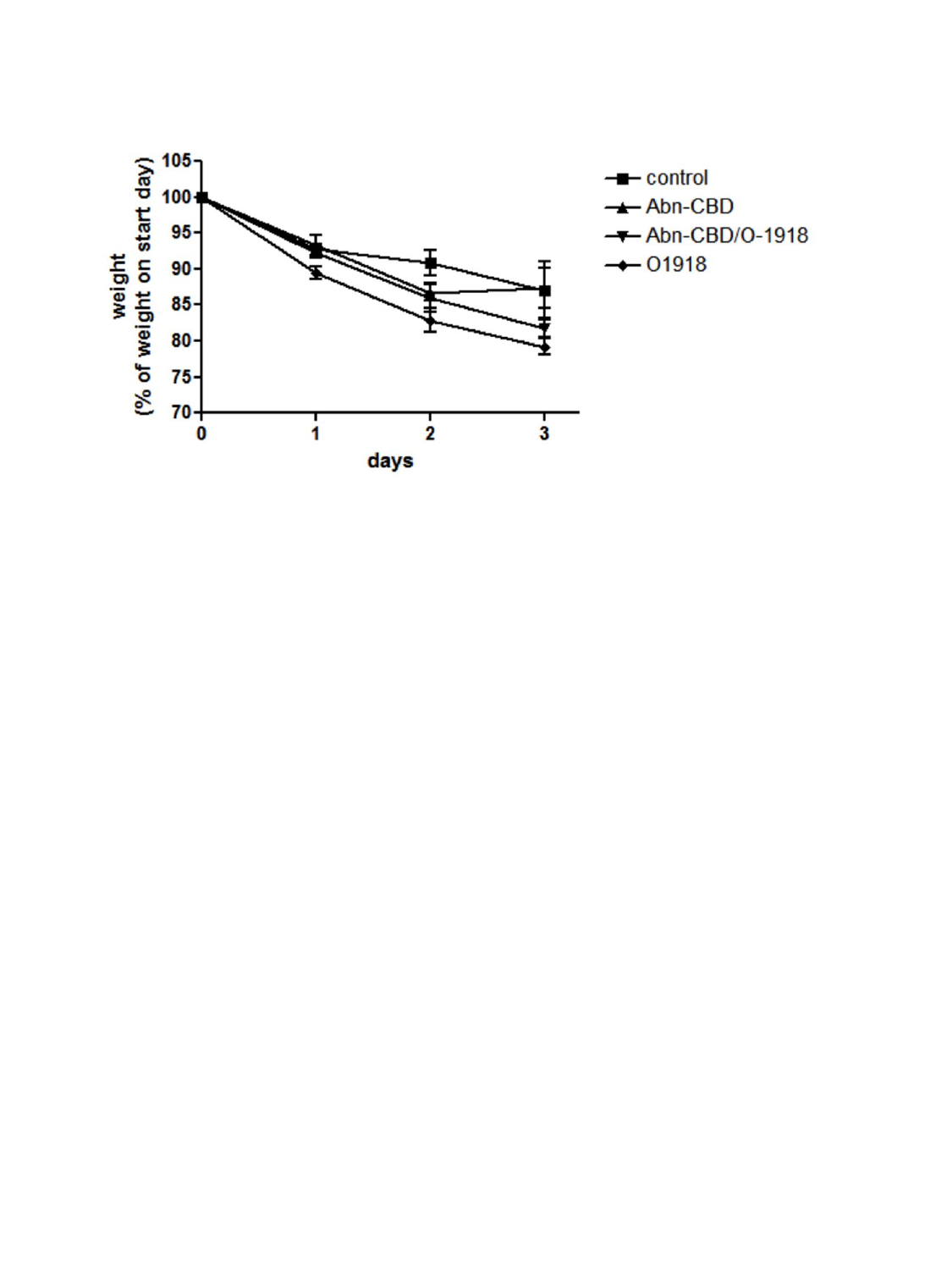

Supplement: Additional file 1: Figure S1 — Weight loss in animals with colitis. O-1918 (5 mg/kg) and/or Abn-CBD (5 mg/kg) were given twice daily for 3 days to TNBS-treated mice. Weight of the animals was recorded daily. All animals lost weight throughout the duration of the experiment. Abn-CBD treatment did not decrease weight loss. No significant differences in weight loss were observed. (PPTX 3237 kb) [file 12950_2016_129_MOESM1_ESM.pptx]
